# Neighborhood-level socioeconomic disadvantage is associated with gut microbial composition and diversity across many chronic disease states

**DOI:** 10.3389/fpubh.2026.1847540

**Published:** 2026-07-01

**Authors:** Josiah E. Radder, Kelvin Li, Melissa Saul, Mehdi Nouraie, Heather Gentry, Asha Patel, Cathy Kessinger, Adam Fitch, Daniel G. Dunlap, Georgios D. Kitsios, Yingze Zhang, Barbara A. Methé, Alison Morris

**Affiliations:** 1 Division of Pulmonary, Allergy, Critical Care, and Sleep Medicine, University of Pittsburgh School of Medicine, Pittsburgh, PA, United States; 2 Center for Medicine and the Microbiome, University of Pittsburgh School of Medicine, Pittsburgh, PA, United States

**Keywords:** chronic disease, gut microbiome, health disparities, microbiome, neighborhood level socioeconomic disadvantage

## Abstract

**Background:**

Socioeconomic disparities play a major role in health and disease. Growing evidence suggests that healthcare access accounts for only part of these outcomes, and additional biological mechanisms remain to be elucidated. The gut microbiome is a central component of health and disease and can be affected by environmental factors and socioeconomic disparities.

**Methods:**

Using a large cohort with diverse comorbidities identified in the Elixhauser comorbidity index, we tested for association between area deprivation index (ADI), a neighborhood-level measurement of socioeconomic disadvantage in the United States, and taxonomic profiles of gut microbiota (16S rRNA gene sequences) to examine the effect of (1) covariates (age, sex, and smoking), ADI, and microbiota on comorbidities and (2) covariates, ADI, and comorbidities on microbiota.

**Findings:**

Covariates explained several associations, and ADI was associated with multiple comorbidities as assessed using generalized linear models (GLMs) augmented with ADI regression splines. Most associations with ADI were nonlinear, and the associations were most frequent among individuals living in more disadvantaged neighborhoods. Multivariate analysis of variance (MANOVA) revealed a significant effect of ADI on the collective microbiota of the cohort. Individual microbial taxa were identified in association with ADI, ranging from potentially more beneficial to human health to more disease-promoting, whereas microbial diversity was negatively associated with over half of the disease associations.

**Interpretation:**

These findings indicate that ADI is associated with alterations to the gut microbiome and common disease states.

## Introduction

Socioeconomic disparities in the United States have been steadily increasing over the past decades, and these disparities have been directly associated with life expectancy (1, 2). This finding is likely driven by the role of socioeconomic status on several major determinants of health, including access to and the quality of health care, lifestyle behaviors, social cohesion, and environmental exposures (3, 4). Socioeconomic status is generally considered a composite of income, education, and occupation, but it is a complex measure and is variably assessed worldwide (5).

The area deprivation index (ADI) is a measure of neighborhood-level disadvantage created by the Health Resources and Services Administration and is calculated from long-form US Census Bureau data. The measure includes factors that are representative of household education, employment, income, and housing quality in a region. Originally generated at the county level, it has since been refined to the neighborhood level by census blocks (6). ADI is associated with clinical outcomes in a range of common diseases, from diabetes to chronic lung disease to cancer (7–9). While many of these studies have identified relationships between healthcare access and socioeconomic status, there are also associations with biological effects of living in a highly disadvantaged neighborhood compared to a less disadvantaged neighborhood, including alterations in epigenetic markers of aging (10). From a health perspective, increases in income (i.e., decreases in socioeconomic disadvantage) may improve access to healthcare, nutritious food, and economic security, thereby improving health outcomes. However, the incremental health gains from additional income are likely to be smaller at higher income levels than at lower ones. This pattern is consistent with the economic concept of “diminishing marginal utility of income,” which states that each additional unit of income provides less added benefit than the previous unit. Consequently, the relationship between socioeconomic deprivation and health outcomes may be nonlinear, with larger effects occurring among more disadvantaged populations.

The human microbiome is a complex collection of trillions of microorganisms that inhabit a human host, collectively carrying three times as many genes as the human genome. Microbiota occupy multiple ecological niches and interact with the host to play major roles in health and disease (11, 12). Each niche occupied by the human microbiome is also exposed in complex ways to environmental factors, many of which may be affected by socioeconomic status. The gut microbiome plays a role in metabolic functions in the human body, shapes the systemic immune response, and has been tied directly to human health and disease. Growing evidence suggests that environmental influences on the gut microbiome may impact the functions of the gut microbiome and contribute to disease (13, 14). Many of the proposed mechanisms through which this occurs, including exposure to environmental chemicals and air pollution, diet, and access to antibiotics, are affected by socioeconomic status (14).

Given the known relationships among socioeconomic disadvantage, disease states, and the biological roles of the gut microbiome, we sought to test the hypothesis that socioeconomic deprivation, measured by ADI, is associated with alterations in gut microbiome and common disease diagnoses. We used a large cohort of individuals with diverse comorbidities to test for associations between gut microbial alpha diversity, individual bacterial taxa, and community composition. We included common comorbidities using an accepted comorbidity score to further identify associations with common human diseases in our models. Associations between ADI (as a predictor) and microbiome features and comorbidities were identified using nonlinear models to account for the potentially stronger effects among more disadvantaged populations.

## Methods

### Cohort

Participants were selected from a large ongoing observational cohort (MedBio), which included individuals with a range of chronic comorbidities (15). Participants were recruited to this cohort directly and from multiple ongoing prospective clinical studies at the University of Pittsburgh Medical Center (UPMC) between 2017 and 2022. All individuals with electronic health record data and a stool sample in these studies were considered eligible. A standard approach to subject enrollment and specimen processing was utilized. The University of Pittsburgh Institutional Review Board approved the study (IRB#19030104), and all participants provided informed consent.

### Sample collection, processing, sequencing, and taxonomic identification

Stool sample collection, processing, sequencing, and taxonomic identification have been previously described and are included in the Supplementary materials (15).

### Matching with the area deprivation index

We identified the home address for all individuals greater than 18 years-old in the MedBio cohort who were recorded during an inpatient or outpatient visit in the UPMC electronic health record. If participants had multiple addresses, the address recorded closest to the time of sample collection was used. All records were processed through the institution’s enterprise master patient index to ensure that subjects with multiple medical record number assignments were assigned only one unique identifier. We then removed all individuals who had not had a visit within 1 year of sample collection. Addresses were coded to census blocks using the US Census Bureau’s geocoding tools as implemented in the R package ‘censusx.’ (16) National ADI scores provided by the Neighborhood Atlas were matched to census blocks, removing individuals living in census blocks without available scores (6). Reported ADI quintiles were as follows: Q1 (1–20), Q2 (21–40), Q3 (41–60), Q4 (61–80), and Q5 (81–100). Analyses were performed on quintiles (rather than quartiles) to provide a finer granularity of ADI stratification. Quintiles also provide a distinct middle stratum (Q3), a meaningful category for the population’s median range of deprivation. Conversely, quartiles split the middle of the population into “lower-middle” and “upper-middle” groups, making the interpretation of the ADI gradient less intuitive.

### Demographic variables and comorbidity index

All diagnosis codes using the International Classification of Diseases (ICD) versions 9 and/or 10 codes recorded in all inpatient or outpatient visits before sample collection were identified in UPMC’s electronic health record (EHR). These were used to code individuals into thirty-two specific comorbidity groups in the Elixhauser comorbidity index and to calculate the overall comorbidity score using the Elixhauser comorbidity index with the R package ‘comorbidity’ (17, 18). Uncomplicated and complicated diabetes or hypertension were grouped into a single classifier of diabetes or hypertension for individual comorbidity analysis and were included independently in the overall index. Gender was self-identified. Age was calculated as the time from sample collection to the participant’s birth date. Smoking status was recorded as either never smoker, former smoker, current smoker, or missing based on curated clinician-recorded responses in the EHR. Age and Elixhauser comorbidity scores are reported as median and interquartile range.

### Statistical analysis preparation

Due to the compositional nature of the taxonomic profiles from 16S rRNA gene sequencing (19), taxonomic abundances were first transformed using the additive log ratio (ALR) transformation (20). The top 25 taxa, by average abundance across the experimental samples, were selected to represent the taxa of interest, and the remaining taxa were accumulated into the denominator of the ratio, before natural log transformation (21).

Analyses involving the calculation of a diversity index utilized the Shannon diversity index and the Tail statistic (22). The Tail statistic is more sensitive to lower-abundance taxa than the Shannon diversity index.

### Model development

**Figure 1:**
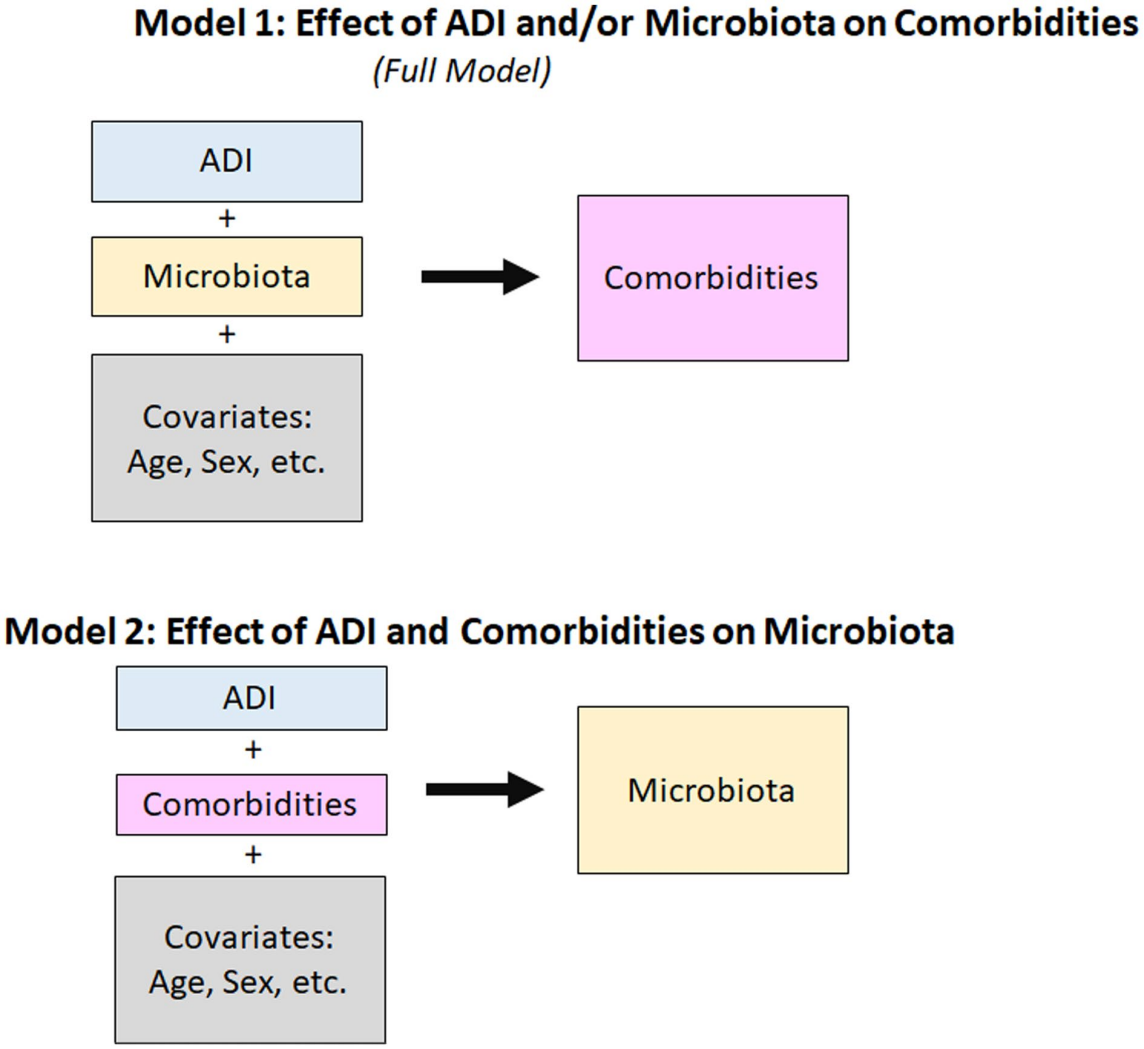
Two models. Model 1: Comorbidities as a Response (CaaR) uses ADI, microbiota, and covariates to predict comorbidities. Model 2: Microbiota as a Response (MaaR) uses ADI, comorbidities, and covariates to predict the microbiota.

Two models were selected to analyze the relationships among the covariates, ADI, comorbidities, and the microbiome. The first model (CaaR: Comorbidities as a Response) used covariates, ADI, and the 16S profile (attributes) as predictors of comorbidities. (Figure 1) The covariates included: sex, age, and recoded smoking status (current smoker and history of smoking). Active smokers were designated as “current,” whereas both active and former smokers were designated with a “history.” This coding attempted to differentiate tobacco smoking’s immediate, exposure-related effects from its cumulative long-term consequences. For both models, the ADI was divided into quintiles, and nonlinear breakpoints (hinges) were applied to each quintile (0, 20, 40, 60, and 80) and included in generalized linear models (GLMs) as covariates. Using this approach, regression splines could capture any significant nonlinear relationships between ADI and comorbidities. The second model (MaaR: Microbiome as a Response) focused on predicting the attributes (ALR and diversity) of the 16S profiles, with the independent variables: covariates, ADI, and comorbidities. The “comorbidities” included the Elixhauser Comorbidity Index, and 29 comorbidities used to calculate this index after combining “uncomplicated” and “complicated” diabetes and hypertension as described in the Methods, which were recorded as Boolean variables (Table 1). In the CaaR models, the 29 Boolean comorbidities were fit with a (binomial family) GLM to perform logistic regressions. Subsequent references to statistical relationships identified between predictor and response variables control for the effects of covariates and ADI. Similarly, the MaaR model controlled for all comorbidities, and the CaaR model controlled for the top 25 taxa for the ALR analyses.

**Table 1:** Description of clinical cohort.

	Quintile 1 (1–20)	Quintile 2 (21–40)	Quintile 3 (41–60)	Quintile 4 (61–80)	Quintile 5 (81–100)
*N*	108	325	446	514	386
Age [median years (IQR)]	61.6 (49.5–71.1)	61 (47.4–70.2)	61.2 (46.9–69.6)	60.7 (48.2–68.7)	57.1 (45.9–66.5)
Sex (% Men)	47.2%	44.9%	36.1%	36.6%	35.8%
Smoking status (never/former/current)	62/27/7	186/95/19	233/143/34	250/176/47	166/127/59
Elixhauser comorbidity score	3 (1–5)	3 (1–6)	4 (2–7)	4 (2.25–8)	5 (2–8)
Congestive heart failure	7 (6.5%)	31 (9.5%)	51 (11.4%)	79 (15.4%)	72 (18.7%)
Cardiac arrhythmias	25 (23.1%)	83 (25.5%)	119 (26.7%)	161 (31.3%)	121 (31.3%)
Cardiac valvular disease	12 (11.1%)	64 (19.7%)	82 (18.4%)	93 (18.1%)	67 (17.4%)
Pulmonary circulation disorders	5 (4.6%)	33 (10.2%)	51 (11.4%)	89 (17.3%)	76 (19.7%)
Peripheral vascular disease	17 (15.7%)	53 (16.3%)	66 (14.8%)	93 (18.1%)	59 (15.3%)
Hypertension	43 (39.8%)	150 (46.2%)	234 (52.5%)	297 (57.8%)	223 (57.8%)
Paralysis	1 (0.9%)	3 (0.9%)	5 (1.1%)	4 (0.8%)	8 (2.1%)
Neurologic disorders	7 (6.5%)	24 (7.4%)	35 (7.8%)	56 (10.9%)	45 (11.7%)
Chronic pulmonary disease	30 (27.8%)	87 (26.8%)	137 (30.7%)	181 (35.2%)	145 (37.6%)
Diabetes	14 (13%)	49 (15.1%)	72 (16.1%)	137 (26.7%)	103 (26.7%)
Hypothyroidism	23 (21.3%)	63 (19.4%)	103 (23.1%)	120 (23.3%)	93 (24.1%)
Renal failure	8 (7.4%)	42 (12.9%)	57 (12.8%)	79 (15.4%)	70 (18.1%)
Liver disease	17 (15.7%)	60 (18.5%)	85 (19.1%)	117 (22.8%)	83 (21.5%)
Peptic ulcer disease	2 (1.9%)	14 (4.3%)	21 (4.7%)	24 (4.7%)	21 (5.4%)
HIV/AIDS	1 (0.9%)	2 (0.6%)	8 (1.8%)	12 (2.3%)	22 (5.7%)
Lymphoma	1 (0.9%)	6 (1.8%)	6 (1.3%)	12 (2.3%)	11 (2.8%)
Metastatic cancer	7 (6.5%)	27 (8.3%)	35 (7.8%)	45 (8.8%)	29 (7.5%)
Solid tumor without metastasis	18 (16.7%)	47 (14.5%)	63 (14.1%)	81 (15.8%)	60 (15.5%)
Rheumatologic disease	16 (14.8%)	74 (22.8%)	113 (25.3%)	146 (28.4%)	123 (31.9%)
Coagulopathy	7 (6.5%)	31 (9.5%)	63 (14.1%)	69 (13.4%)	55 (14.2%)
Obesity	20 (18.5%)	96 (29.5%)	139 (31.2%)	173 (33.7%)	169 (43.8%)
Weight loss	13 (12%)	52 (16%)	73 (16.4%)	110 (21.4%)	80 (20.7%)
Fluid and electrolyte disorders	27 (25%)	92 (28.3%)	130 (29.1%)	185 (36%)	145 (37.6%)
Blood loss anemia	4 (3.7%)	13 (4%)	17 (3.8%)	20 (3.9%)	20 (5.2%)
Deficiency anemia	9 (8.3%)	58 (17.8%)	75 (16.8%)	106 (20.6%)	83 (21.5%)
Alcohol use disorder	0 (0%)	8 (2.5%)	12 (2.7%)	25 (4.9%)	21 (5.4%)
Drug use disorder	3 (2.8%)	16 (4.9%)	18 (4%)	26 (5.1%)	44 (11.4%)
Psychoses	0 (0%)	0 (0%)	2 (0.4%)	8 (1.6%)	3 (0.8%)
Depression	28 (25.9%)	91 (28%)	143 (32.1%)	181 (35.2%)	156 (40.4%)

Individuals are separated by national ADI quintiles. For the individual comorbidities, the number of individuals with a specific comorbidity in each quintile is displayed with the percentage of the overall quintile in parentheses.

Briefly, linear splines are a type of nonlinear regression in which a series of hinge (break) points are defined for the target predictor (e.g., ADI), allowing multiple slopes (coefficients) to be estimated for each segment bounded by the hinge points. In our analysis, we use the evenly divided quintiles (5 segments). These hinge points were introduced into a standard linear regression as 5 new variables: ADI_00, ADI_20, ADI_40, ADI_60, and ADI_80. The formula used for each new ADI_X variable is max (0, ADI-X). Thus, ADI_00 contains the same values as the original ADI variable, but ADI_10 through ADI_80 have a “hockey stick” shape. The hinge point is the heel of the blade of a hockey stick. Since linear regression is additive across all predictors, the combination of all the ADI_X estimates can reveal when ADI begins to associate with disease outcome (slopes become non-zero) and/or if the association discontinues or worsens at higher ADI levels. For example, for drug misuse, ADI_00 through ADI_40 all had essentially 0 slopes, but at ADI_60, a positive slope was identified. This suggests drug misuse was not associated with socioeconomic deprivation until the 60th quintile, at which point worsening deprivation was associated with increasing drug misuse.

## Results

### Description of clinical cohort

**Figure 2:**
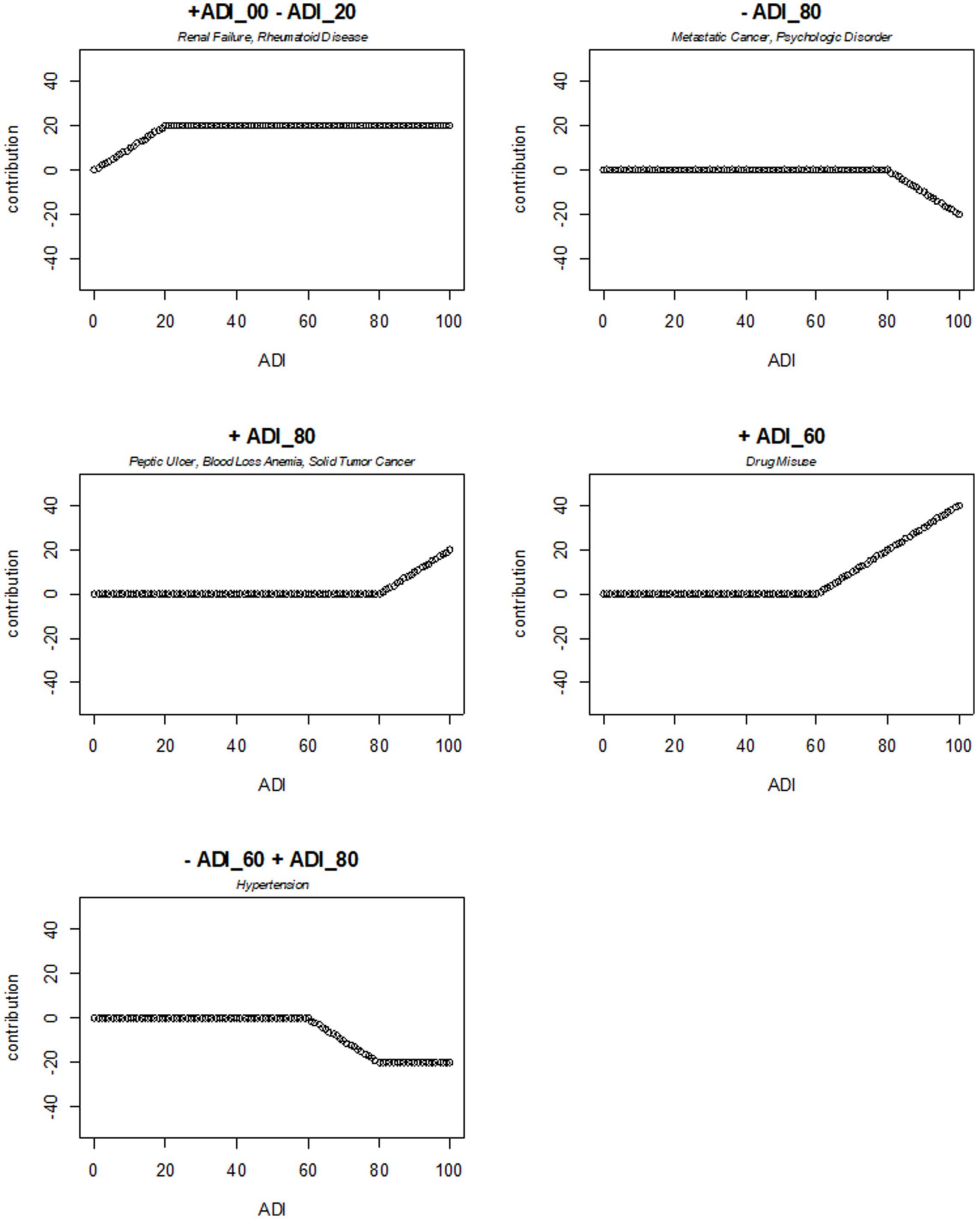
Patterns of nonlinear associations between ADI and specific comorbidities.

**Figure 3:**
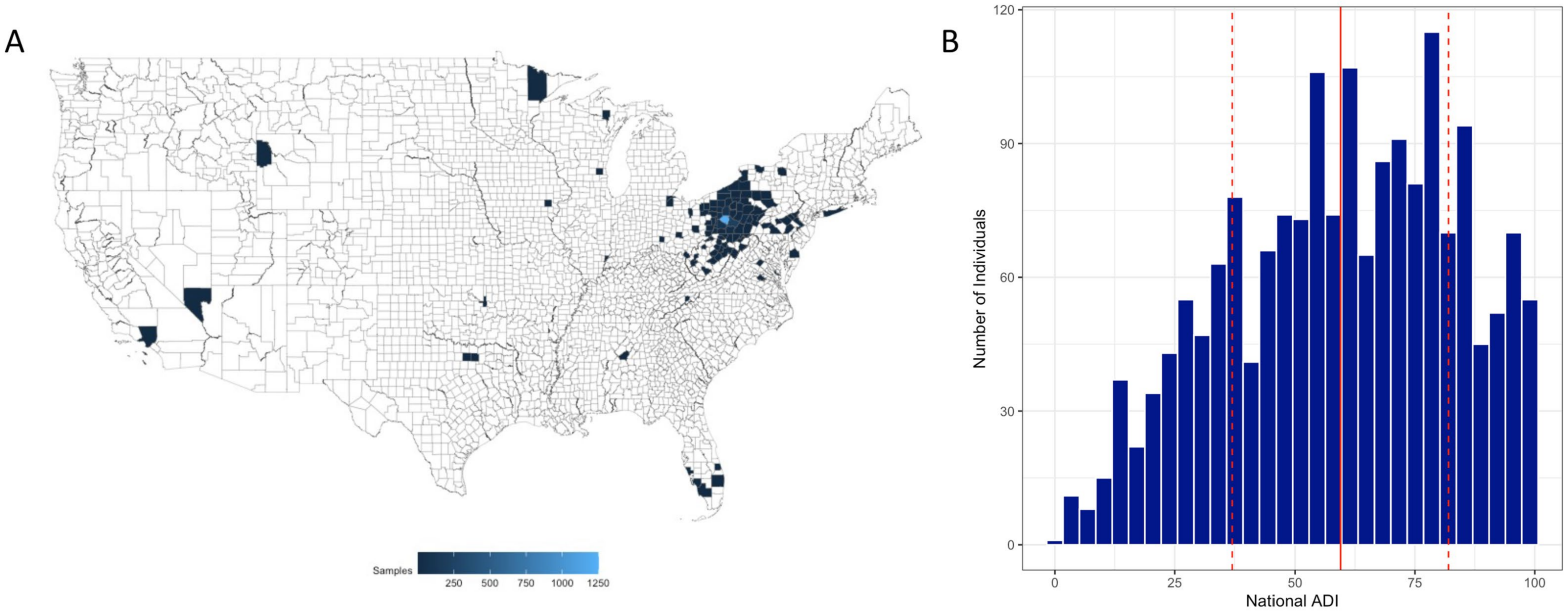
Individuals in the study cohort were primarily from western Pennsylvania, West Virginia, and Ohio, and the average area deprivation is higher than the national average. **(A)** Chloropleth of county-level counts of individuals included in this study. **(B)** Histogram of counts of individuals binned by national area deprivation index, with a solid red line representing the population mean (compared to the national mean of 50) and dotted red lines representing the first and fifth quintiles of the population.

We identified a total of 1,779 individuals with stool microbiome samples who had a visit documented in the electronic health record within 1 year and an address that could be coded in the US Census Bureau datasets and matched with ADI (Figure 2). The median national ADI of our cohort was slightly higher than the national average (61.0, IQR 42.0–78.0) (Figure 3, Table 1).

No clear relationship was observed between ADI and age in the cohort; however, more disadvantaged individuals were more likely to be women (Quintile 1: 53%, Quintile 5: 64%), more likely to be current or former smokers (Quintile 1: 31%, Quintile 5: 65%), and had higher Elixhauser comorbidity scores [Quintile 1: 3 (1–5), Quintile 5: 5 (2–8)] than less disadvantaged individuals (Table 1). Moreover, several individual comorbidities were more prevalent among more disadvantaged individuals, notably congestive heart failure (Quintile 1: 6.5%, Quintile 5: 18.7%), pulmonary circulation disorders (Quintile 1: 4.6%, Quintile 5: 19.7%), hypertension (Quintile 1: 39.8%, Quintile 5: 57.8%), diabetes mellitus (Quintile 1: 13%, Quintile 5: 16.7%), HIV/AIDS (Quintile 1: 0.9%, Quintile 5: 5.7%), rheumatologic disease (Quintile 1: 14.8%, Quintile 5: 31.9%), and obesity (Quintile 1: 18.5%, Quintile 5: 43.8%).

## Comorbidities as a Response (CaaR)

### ADI and microbiota are associated with comorbidities when modeling Comorbidities as a Response (CaaR)

**Figure 4:**
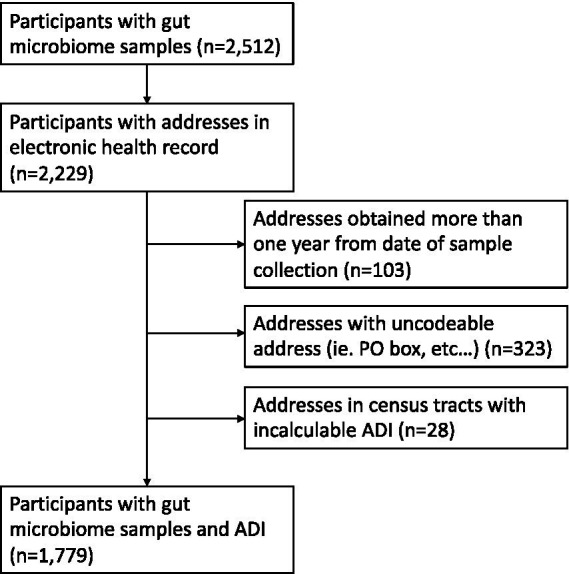
Identification of area deprivation index (ADI) in MedBio cohort. The addresses of participants in MedBio with gut microbiome samples were identified; addresses older than 1 year from sample collection, and uncodeable addresses were removed, resulting in a final population of 1,779 participants.

Overall, when integrating microbiota by controlling for specific taxonomic abundances (ALR), ADI predicted multiple comorbidities (Figure 4). Distinct patterns of associations (i.e., combinations of segments with significant non-zero slopes) were identified for the various comorbidities.

Renal failure and rheumatologic disorders both necessitated 2 hinges (positive at ADI-00 and negative at ADI-20, with *p* < 0.1). This dual-hinge fit indicates a positive association within the first ADI quintile (0–20%), but after reaching the second quintile (20%), there was no further increase in reported disease level in the remaining quintiles (20–100%) (Figure 4). Metastatic cancer and mental health disorders had no associations with ADI until the last quintile (ADI-80 hinge, *p* < 0.05), when a negative association began there. Conversely, the following comorbidities had positive associations at the last quintile: peptic ulcer disease (*p* < 0.05), blood loss anemia (*p* < 0.05), and solid tumor cancers (*p* < 0.1). These results indicate that for these comorbidities, deprivation is not associated with higher or lower reporting levels until the most-deprived 5th quintile. Drug misuse had a positive association (*p* < 0.05) from the 4th to the final quintile (ADI-60, *p* < 0.05). Hypertension also necessitated a dual hinge fit. The combination of association at ADI-60 (negative, *p* < 0.1) and at ADI-80 hinge (positive, *p* < 0.05) revealed that deprivation was not associated with hypertension reports until the fourth quintile (60–80%). At the 4th quintile, the association between ADI and hypertension was negative until the final quintile, when the reported hypertension did not continue to associate negatively with worsening deprivation. All individual associations are reported in Figure 2. As demonstrated in this paragraph, when multiple ADI hinges are associated with a comorbidity, the net effect at a specific ADI value is estimated by accumulating the ADI_X coefficients below that target value. For example, to identify the association between ADI and Hypertension at ADI = 70, only the negative association of the ADI_60 hinge point needs to be considered, but at ADI = 85, both the ADI_60 and ADI_80 hinge points need to be accumulated to interpret that the association is negative, but no longer worsening at greater ADI values.

### Covariates were associated with multiple comorbidities (CaaR)

Age was negatively associated with drug use disorder and mental health disorders. No associations with age were identified for HIV/AIDS, fluid and electrolyte disorders, blood loss anemia, deficiency anemia, and alcohol use disorder. All other comorbidities, including the Elixhauser index, were positively associated with age. Current smoking was positively associated with alcohol, paralysis, mental health disorders, and HIV/AIDS.

### Alpha diversity predicts multiple comorbidities (CaaR)

When the microbiota was represented in the models as diversity and a predictor, statistically significant negative associations were identified between microbial diversity and more than half of the comorbidities (diabetes, fluid and electrolyte disorders, deficiency anemias, chronic pulmonary disease, liver disease, solid tumors, obesity, depression, peptic ulcer disease, pulmonary circulation disorders, congestive heart failure, drug use disorder, hypertension, weight loss, and coagulopathies) (Supplementary Figure 4). Utilizing the Tail statistic, microbial diversity was also significantly associated with HIV/AIDS (*p* < 0.01, positive). Alpha diversity was not a significant predictor of the remaining comorbidities. Decreased alpha diversity was also a significant predictor of a higher Elixhauser index.

**Figure 5:**
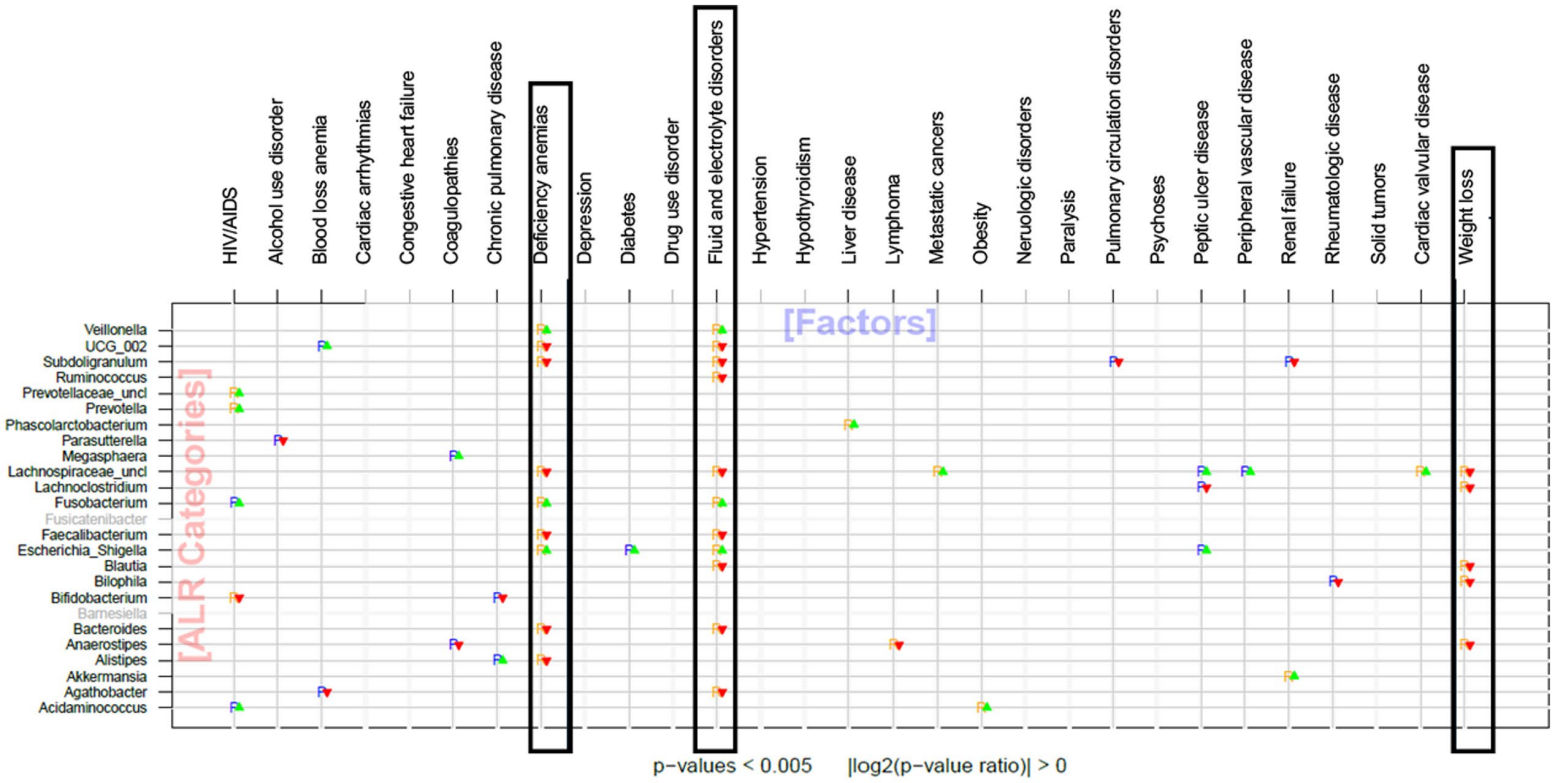
Predictor responder analysis. A “P” indicates that the taxon (row) predicts the column (comorbidity). An “R” indicates that the taxon (row) responds to the column (comorbidity). A green upward-pointing triangle indicates that the association is positive. A red downward-pointing triangle indicates that the association is negative.

ADI hinges were significantly associated with comorbidities when diversity (Tail and Shannon) was included as a predictor in the models. The same general pattern of associations was identified as earlier described when the CaaR model used the microbiota abundance (ALR) in place of diversity (Figure 5).

### Microbial taxa demonstrate a range of possible health and disease predictions (CaaR)

Most individual taxa displayed a range of associations in terms of the number and strength of these associations (as indicated by *p*-values) and directionality (positive or negative) with disease, suggesting greater complexity in their ecological roles (Table 2). Along this continuum, several taxa were identified with mostly negative associations with multiple comorbidities, suggesting that these taxa may be more benign or protective against disease or may be markers of better health. *Subdoligranulum* was identified with the most significant number of associations, all of which were negative. Additional taxa identified with more than six negative associations with specific comorbidities were *Anaerostipes*, *Bifidobacterium*, *Faecalibacterium*, *Lachnoclostridium,* and *Barnesiella* (*p* < 0.1). Several taxa were identified as positively associated with multiple comorbidities, suggesting that these taxa may promote or serve as markers of disease. The taxa identified with six or more total significant positive associations (*p* < 0.1) were *Lachnospiraceae_unclassified* (8 associations), *Alistipes* (6 associations), and *Escherichia_Shigella* (13 associations). *Escherichia* and *Shigella*, with 13 positive associations, are particularly noteworthy; their only negative association is with psychoses (*p*-value < 0.05).

**Table 2:** Microbial taxa demonstrate a range of disease prediction.

Genus	Phylum	Class	“Protective” (negative associations with comorbidities)	“Disease promoting” (positive associations with comorbidities)
Subdoligranulum	Firmicutes	Oscillospiraceae	9	0
Anaerostipes	Firmicutes	Lachnospiraceae	8	1
Bifidobacterium	Actinobacteria	Bifidobacteriaceae	7	2
Faecalibacterium	Firmicutes	Ruminococcaceae	7	1
Barnesiella	Bacteroidetes	Barnesiellaceae	6	2
Lachnoclostridium	Firmicutes	Lachnospiraceae	6	1
Parasutterella	beta-proteobacteria	Sutterellaceae (Burks)	5	3
Agathobacter	Firmicutes	Lachnospiraceae	4	1
Bilophila	delta-proteobacteria	Desulfovibrionaceae	4	1
UCG_002	Firmicutes	Clostridia?	4	1
Blautia	Firmicutes	Lachnospiraceae	3	1
Fusicatenibacter	Firmicutes	Lachnospiraceae	3	0
Akkermansia	Verrucomicrobia	Verrucomicrobiales	2	2
Lachnospiraceae_uncl	Firmicutes	Lachnospiraceae	0	8
Alistipes	Bacteroidetes	Rikenellaceae	0	6
Acidaminococcus	Firmicutes	Acidaminococcaceae (Negativicutes)	0	4
Prevotellaceae_uncl	Bacteroidetes	Prevotellaceae	0	4
Veillonella	Firmicutes	Veillonellaceae (Negativicutes)	0	3
Fusobacterium	Fusobacteriota	Fusobacteriaceae	0	2
Megasphaera	Firmicutes	Veillonellaceae (Negativicutes)	0	2
Escherichia_Shigella	gamma-proteobacteria	Enterobacteraceae	1	14
Bacteroides	Bacteroidetes	Bacteroidaceae	1	5
Phascolarctobacterium	Firmicutes	Acidaminococcaceae (Negativicutes)	1	3
Ruminococcus	Firmicutes	Oscillospiraceae	1	0
Prevotella	Bacteroidetes	Prevotellaceae	2	6

Numbers of “protective” and “disease-promoting” associations are reported for each taxon. Orange highlighted taxa have six or more protective associations, while green highlighted taxa have 6 or more disease promoting associations.

Four taxa were negatively associated with increasing Elixhauser score: *Anaerostipes*, *Barnesiella*, *Faecalibacterium,* and *Subdoligranulum*. Five taxa were positively associated with increasing Elixhauser score: *Acidaminococcus*, *Alistipes*, *Escherichia-Shigella*, *Lachnospiraceae_uncl*, and *Prevotellaceae_uncl*.

## Microbiome as a Response (MaaR)

### Age and several comorbidities predict alpha diversity (MaaR)

There was no significant effect of ADI on alpha diversity in Model 2 (MaaR) except for a positive association of the ADI-80 hinge with the Tail statistic (coef = 0.017, *p* = 0.077). The Elixhauser score was not identified as a significant predictor of diversity. Age was positively associated with alpha diversity (Tail and Shannon) (Tail, coef = 0.008, *p* = 4.45e−06). Significant positive associations were also identified with HIV/AIDS, metastatic cancer, and rheumatological disorders, with diversity (Tail and Shannon). Alcohol use disorder was also predictive of greater Shannon diversity.

### ADI had a significant effect on the collective microbiota (MaaR)

From multivariate analysis of variance (MANOVA), there was a significant association between the microbiota and two hinges (ADI-00 and ADI-60). Age, sex, and current smoking status were identified as having a significant effect on the microbiota. The covariate of smoking history was not significantly associated with gut microbiota. However, it was associated with the comorbidities according to the CaaR model (Model 1), suggesting that smoking history may affect the host more than the microbiota. Results from distance-based PERMANOVA (beta-diversity) were generally consistent with the abundance-based MANOVA and were therefore not reported.

Results from the underlying univariate analyses found significant, predictive associations (*p* < 0.05) of ADI hinges only with *Bilophila*, a delta-proteobacterium and sulfate reducer, and *Parasutterella*, an asaccharolytic and secondary-bile-acid-producing beta-proteobacterium. For *Bilophila,* the ADI-40 (coef = −0.07) and ADI-80 (coef = −0.05) hinges had a negative association, while the ADI-60 hinge (coef = 0.06) had a positive association with the abundance of *Bilophila*. This combination of hinges can be interpreted as no association with deprivation until the 40th percentile (−0.07), weaker negative association starting from the 60th percentile (−0.07 + 0.06 = −0.01), then continued stronger negative association after the 80th percentile (−0.07 + 0.06 + −0.05 = −0.06). If fewer hinge points had been utilized (e.g., quartiles instead of quintiles), a single hinge point at 50% might have been significant. For *Parasutterella*, the hinge point combination of ADI-20 (positive, coef = 0.12) and ADI-40 (negative, coef = −0.06) suggests an increase of *Parasutterella* with disadvantage starting at the 20th percentile, then a slight weakening, while still increasing (0.12 + −0.06 = 0.06) association from the 40th percentile and thereafter.

## Discussion

This study demonstrates associations between a measure of neighborhood-level socioeconomic status and both the diversity and composition of the gut microbiome, as well as common disease states. Using two models, we examined the effect of ADI and gut microbiota on comorbidities (CaaR) and the effect of ADI and comorbidities on gut microbiota (MaaR).

When standard covariates of age, sex, and smoking were included in both models, these variables proved more informative than ADI in explaining effects on either comorbidities or the microbiota (represented via abundance or diversity). We found that using ADI to predict comorbidities can be nonlinear [Model 1 (CaaR)]. When significant, these inflection points, where the rates of association with comorbidities change, occur more frequently at higher (more disadvantaged) ADI scores. Similar to our work, an examination of ADI (used as a dichotomized median in linear models) and gut microbiota interactions in relation to cognitive impairment among individuals with cirrhosis suggested that demographics and cirrhosis-related variables may be more influential on gut microbiota and cognitive impairment than ADI in multivariable analyses (23). However, the application of a systematic statistical framework and the augmentation of the GLM models with ADI hinges (to perform regression splines) in the current study were beneficial in deepening this understanding by recognizing that these associations may be nonlinear.

When microbial diversity was used as a predictor, it was negatively associated with more than half of the comorbidities in our study. Decreased alpha diversity has previously been found to be associated with common disease states, including diabetes, obesity, and heart failure, in both large cohort studies of the gut microbiome and in individual disease states (24–28). Notably, many of the common diseases included as comorbidities in the Elixhauser index have previously been associated with alterations in the gut microbiome (25, 29, 30). Our findings are consistent with this prior work, as the Elixhauser index was also associated with decreased gut microbial diversity in this study. Further, our work identified several comorbidities that were not associated with alterations to gut microbial alpha diversity. ADI predicted alpha diversity in Model 1 when controlling for age and gender, whereas exposure to greater neighborhood-level socioeconomic disadvantage (Model 2) did not significantly affect alpha diversity. These findings suggest that neighborhood-level socioeconomic disadvantage does not independently drive changes in gut microbial alpha diversity; rather, this association is in part mediated by an increase in diversity-altering comorbidities.

Microbial composition is also associated with neighborhood-level socioeconomic disadvantage and specific comorbidities. Using MANOVA, we found a significant association between ADI (at all levels) and the overall microbiome of the cohort. We also found that individual taxa within the microbiota may exist on a continuum, ranging from more “protective” or benign to potentially more “disease-promoting” states. Taxa identified as potentially more protective versus disease-promoting are phylogenetically diverse. However, “protective” taxa included several that have been previously identified as potentially beneficial to human health, including *Bifidobacterium* and *Faecalibacterium* (31, 32). *Subdoligranulum*, which had the most negative associations with comorbidities in our study, has been identified as a marker of metabolic health (33). Conversely, more disease-promoting taxa identified in this study included *Escherichia-Shigella,* which has previously been associated with multiple diseases (34). Our analysis could not categorize specific taxa as “protective” versus “disease-promoting,” but the taxa identified could serve as markers of health or disease. Taken together, alterations in overall gut microbial composition that are associated with neighborhood-level socioeconomic disadvantage could also be driven by differences in disease prevalence between these neighborhoods, even though MaaR models controlled for ADI, covariates, and comorbidities.

These results demonstrate that both gut microbial diversity and composition are associated with neighborhood-level socioeconomic disadvantage. Not surprisingly, exposure to disadvantage does not drive these findings independently; instead, it appears to affect overall health status, including bidirectional interactions between the host and microbiome. We know that major determinants of health, such as health care access and quality, lifestyle behaviors, and environmental exposures, affect both the prevalence of individual disease states and overall health. These findings further solidify the need for public health measures that can intervene in response to disparities between neighborhoods (3, 4). Furthermore, evidence that the combined effect of comorbidities, which are more prevalent in disadvantaged neighborhoods, alters the gut microbiome suggests that interventions targeting the gut microbiome may still play a role in chronic disease and possibly in overall health.

Our study has several limitations. As previously discussed, socioeconomic status likely mediates biological outcomes through complex interactions involving both human behavior and environmental exposures that are challenging to measure directly. ADI is a neighborhood-level measure applied to individuals and is calculated using the US Census Bureau data, which may have changed at the time an individual sample is collected. Furthermore, some geographic areas may exhibit greater socioeconomic heterogeneity, so the ADI assigned to a subject may differ meaningfully from individual-level deprivation. Furthermore, race was excluded as a variable in this study. Because race and socioeconomic deprivation are closely intertwined, modeling ADI already captures a portion of the pathways through which race-associated disparities influence health outcomes. Inclusion of race in this context could therefore obscure the deprivation-related mechanisms under investigation. Nevertheless, race may still represent additional biological, cultural, behavioral, environmental, or ancestry-related factors not fully captured by ADI. Future studies may benefit from directly modeling these more specific variables rather than race itself. This study was conducted primarily among individuals already participating in prospective clinical trials in our healthcare system, which may introduce selection bias, particularly toward individuals with better access to healthcare. However, this would most likely lead to underrepresentation of those individuals with the highest socioeconomic disadvantage rather than the least. Due to the breadth of comorbidities examined in this study, it was not possible to statistically control for comprehensive disease-specific variables. While this could potentially strengthen the associations with ADI at specific thresholds (hinge points), it could also provide a stronger physiological explanation for the disease that ADI was only a proxy for. Furthermore, given the breadth of the study, associations were not excluded solely because they failed to meet statistical significance thresholds after correction for multiple testing. We sought to avoid treating multiple-testing-adjusted significance as a binary criterion for reporting results, instead providing the full set of observed associations so that investigators may interpret and prioritize findings in the context of domain-specific knowledge and existing evidence. Nonetheless, we believe the results of this study provide a valuable resource not only for hypothesis generation but also as a roadmap for future analyses of similar scope and complexity.

## Conclusion

Neighborhood-level socioeconomic disadvantage, as measured by ADI, is associated with increased comorbidity prevalence and alterations to gut microbial community composition. The association between ADI and comorbidities is nonlinear, and its effect on overall gut microbial composition is more pronounced than at the level of individual taxa. ADI predicts alpha diversity, but alpha diversity does not independently respond to ADI, suggesting that neighborhood-level differences in age, gender, and comorbidities drive this association. Deconvoluting these relationships will require further studies in large populations with refined clinical phenotypes, individual socioeconomic variables, and the incorporation of analytical approaches that extend beyond linear models. However, our study suggests that novel approaches to disease treatment or prevention that include microbiome modifications can contribute to health-related disparities.

## Data Availability

The sequence data supporting conclusions in this article are available at the National Center for Biotechnology Information (NCBI) BioProject database under BioProject accession number PRJNA1479048.

## References

[1] Bor J Cohen GH Galea S. Population health in an era of rising income inequality: USA, 1980-2015. Lancet. (1980) 389:1475–90. doi: 10.1016/s0140-6736(17)30571-8 28402829

[2] Meara ER Richards S Cutler DM. The gap gets bigger: changes in mortality and life expectancy, by education, 1981-2000. Health Aff (Millwood). (2008) 27:350–60. doi: 10.1377/hlthaff.27.2.350, 18332489 PMC2366041

[3] Adler NE Newman K. Socioeconomic disparities in health: pathways and policies. Health Aff (Millwood). (2002) 21:60–76. doi: 10.1377/hlthaff.21.2.60, 11900187

[4] Wilson DK Kirtland KA Ainsworth BE Addy CL. Socioeconomic status and perceptions of access and safety for physical activity. Ann Behav Med. (2004) 28:20–8. doi: 10.1207/s15324796abm2801_4, 15249256

[5] Psaki SR Seidman JC Miller M Gottlieb M Bhutta ZA Ahmed T . Measuring socioeconomic status in multicountry studies: results from the eight-country MAL-ED study. Popul Health Metrics. (2014) 12:8. doi: 10.1186/1478-7954-12-8, 24656134 PMC4234146

[6] Kind AJH Buckingham WR. Making neighborhood-disadvantage metrics accessible - the neighborhood atlas. N Engl J Med. (2018) 378:2456–8. doi: 10.1056/NEJMp1802313, 29949490 PMC6051533

[7] Galiatsatos P Woo H Paulin LM Kind A Putcha N Gassett AJ . The association between neighborhood socioeconomic disadvantage and chronic obstructive pulmonary disease. Int J Chron Obstruct Pulmon Dis. (2020) 15:981–93. doi: 10.2147/COPD.S238933, 32440110 PMC7211318

[8] Kurani SS Lampman MA Funni SA Giblon RE Inselman JW Shah ND . Association between area-level socioeconomic deprivation and diabetes care quality in US primary care practices. JAMA Netw Open. (2021) 4:e2138438-e. doi: 10.1001/jamanetworkopen.2021.38438, 34964856 PMC8717098

[9] Yu K-X Yuan W-J Huang C-H Xiao L Xiao R-S Zeng P-W . Socioeconomic deprivation and survival outcomes in patients with colorectal cancer. Am J Cancer Res. (2022) 12:829–38. 35261805 PMC8899994

[10] Lawrence KG Kresovich JK O'Brien KM Hoang TT Xu Z Taylor JA . Association of neighborhood deprivation with epigenetic aging using 4 clock metrics. JAMA Netw Open. (2020) 3:e2024329. doi: 10.1001/jamanetworkopen.2020.24329, 33146735 PMC7643028

[11] Sender R Fuchs S Milo R. Revised estimates for the number of human and Bacteria cells in the body. PLoS Biol. (2016) 14:e1002533. doi: 10.1371/journal.pbio.1002533, 27541692 PMC4991899

[12] Gevers D Knight R Petrosino JF Huang K McGuire AL Birren BW . The human microbiome project: a community resource for the healthy human microbiome. PLoS Biol. (2012) 10:e1001377. doi: 10.1371/journal.pbio.1001377, 22904687 PMC3419203

[13] Nicholson JK Holmes E Kinross J Burcelin R Gibson G Jia W . Host-gut microbiota metabolic interactions. Science. (2012) 336:1262–7. doi: 10.1126/science.1223813, 22674330

[14] Tu P Chi L Bodnar W Zhang Z Gao B Bian X . Gut microbiome toxicity: connecting the environment and gut microbiome-associated diseases. Toxics. (2020) 8. doi: 10.3390/toxics8010019, 32178396 PMC7151736

[15] Li K Methé BA Fitch A Gentry H Kessinger C Patel A . Gut and oral microbiota associations with viral mitigation behaviors during the COVID-19 pandemic. Front Cell Infect Microbiol. (2022) 12:966361. doi: 10.3389/fcimb.2022.966361, 36159641 PMC9500509

[16] Prener CG Fox B. Creating open source composite geocoders: pitfalls and opportunities. Trans GIS. (2021) 25:1868–87. doi: 10.1111/tgis.12741

[17] Gasparini A. Comorbidity: an R package for computing comorbidity scores. J Open Source Softw. (2018) 3. doi: 10.21105/joss.00648

[18] Elixhauser A Steiner C Harris DR Coffey RM. Comorbidity measures for use with administrative data. Med Care. (1998) 36:8–27. doi: 10.1097/00005650-199801000-00004, 9431328

[19] Gloor GB Macklaim JM Pawlowsky-Glahn V Egozcue JJ. Microbiome datasets are compositional: and this is not optional. Front Microbiol. (2017) 8. doi: 10.3389/fmicb.2017.02224, 29187837 PMC5695134

[20] Aitchison J. The statistical analysis of compositional data. J R Stat Soc B. (1982) 44:139–60. doi: 10.1111/j.2517-6161.1982.tb01195.x

[21] Tarabichi Y Li K Hu S Nguyen C Wang X Elashoff D . The administration of intranasal live attenuated influenza vaccine induces changes in the nasal microbiota and nasal epithelium gene expression profiles. Microbiome. (2015) 3:74. doi: 10.1186/s40168-015-0133-2, 26667497 PMC4678663

[22] Li K Bihan M Yooseph S Methé BA. Analyses of the microbial diversity across the human microbiome. PLoS One. (2012) 7:e32118. doi: 10.1371/journal.pone.0032118, 22719823 PMC3374608

[23] Bajaj JS Fagan A McGeorge S Sterling RK Rogal S Sikaroodi M . Area deprivation index and gut-brain axis in cirrhosis. Clin Transl Gastroenterol. (2022) 13:e00495. doi: 10.14309/ctg.0000000000000495, 35537854 PMC9236605

[24] Beale AL O’Donnell JA Nakai ME Nanayakkara S Vizi D Carter K . The gut microbiome of heart failure with preserved ejection fraction. J Am Heart Assoc. (2021) 10:e020654. doi: 10.1161/JAHA.120.020654, 34212778 PMC8403331

[25] Sun W Du D Fu T Han Y Li P Ju H. Alterations of the gut microbiota in patients with severe chronic heart failure. Front Microbiol. (2021) 12:813289. doi: 10.3389/fmicb.2021.813289, 35173696 PMC8843083

[26] Maskarinec G Raquinio P Kristal BS Setiawan VW Wilkens LR Franke AA . The gut microbiome and type 2 diabetes status in the multiethnic cohort. PLoS One. (2021) 16:e0250855. doi: 10.1371/journal.pone.0250855, 34161346 PMC8221508

[27] Jackson MA Verdi S Maxan ME Shin CM Zierer J Bowyer RCE . Gut microbiota associations with common diseases and prescription medications in a population-based cohort. Nat Commun. (2018) 9:2655. doi: 10.1038/s41467-018-05184-7, 29985401 PMC6037668

[28] Lloyd-Price J Abu-Ali G Huttenhower C. The healthy human microbiome. Genome Med. (2016) 8:51. doi: 10.1186/s13073-016-0307-y, 27122046 PMC4848870

[29] Avery EG Bartolomaeus H Maifeld A Marko L Wiig H Wilck N . The gut microbiome in hypertension. Circ Res. (2021) 128:934–50. doi: 10.1161/CIRCRESAHA.121.318065, 33793332

[30] Chen Y Lin H Cole M Morris A Martinson J Mckay H . Signature changes in gut microbiome are associated with increased susceptibility to HIV-1 infection in MSM. Microbiome. (2021) 9:237. doi: 10.1186/s40168-021-01168-w, 34879869 PMC8656045

[31] Arboleya S Watkins C Stanton C Ross RP. Gut Bifidobacteria populations in human health and aging. Front Microbiol. (2016) 7. doi: 10.3389/fmicb.2016.01204, 27594848 PMC4990546

[32] Martín R Rios-Covian D Huillet E Auger S Khazaal S Bermúdez-Humarán LG . Faecalibacterium: a bacterial genus with promising human health applications. FEMS Microbiol Rev. (2023) 47. doi: 10.1093/femsre/fuad039, 37451743 PMC10410495

[33] Van Hul M Le Roy T Prifti E Dao MC Paquot A Zucker J-D . From correlation to causality: the case of *Subdoligranulum*. Gut Microbes. (2020) 12:1849998–13. doi: 10.1080/19490976.2020.1849998, 33323004 PMC7744154

[34] Anderson M Sansonetti PJ Marteyn BS. Shigella diversity and changing landscape: insights for the twenty-first century. Front Cell Infect Microbiol. (2016) 6. doi: 10.3389/fcimb.2016.00045, 27148494 PMC4835486

