# Supplementary material for: Neighborhood-level socioeconomic disadvantage is associated with gut microbial composition and diversity across many chronic disease states
Source: Front Public Health. 2026 Jul 1;14:1847540. doi: 10.3389/fpubh.2026.1847540 (PMC13368754; doi:10.3389/fpubh.2026.1847540)

Supplemental materials

***Methods***

*Sample collection*

Stool specimens were self-collected using the DNA/RNA Shield Fecal Collection tubes (Zymo) for nucleic acid preservation and short-term (two to four weeks) storage at ambient temperature. Specimens were mailed to the University of Pittsburgh. Upon receipt, specimens were aliquoted prior to long-term storage at -80°C.

*DNA extraction*

DNA extraction was performed using the Qiagen Powersoil Microbiome Kit EP for automated DNA extraction using an Eppendorf, 5075VTC liquid handling workstation. HEPA filtration was used during sample processing, and the workstation was UV-sanitized between batches. Specimens were processed per manufacturer’s protocol with the following modifications: An approximate aliquot of 300μl of specimen was added to individual bead beating tubes to ensure no carryover between samples during the bead beating process. Aliquots from the individual tubes were then transferred to 96-well blocks for completion of the automated genomic DNA extraction process. Reagent blanks were included as negative controls. Cells and genomic DNA from a microbial community of known composition (ZymoBiomics Microbial Community Standards; Zymo Research, Irvine, CA) served as positive controls. As a component of the QC process, positive controls were evaluated across sample batches to evaluate laboratory and sequencing performance and compared to historical performance of 16S rRNA gene sequencing at the Center for Medicine and the Microbiome (CMM). No significant batch deviation was identified.

*Bacterial community sequencing*

Extracted genomic DNA (gDNA) was amplified for the V4 region using Q5 HS High‐Fidelity polymerase (New England BioLabs, Ipswich, MA) with inline barcode primers design as previously described (*15, 16*). V4 primer sequences were: 515f 5’-GTGCCAGCMGCCGCGGTAA-3’ and 806r 5’-GGACTACHVGGGTWTCTAAT-3’. Approximately 5-10 ng of each sample were amplified in 25 µL reactions. Cycle conditions were 98°C for 30 seconds, then 30 cycles of 98°C for 10 seconds, 57°C for 30 seconds, and 72°C for 30 seconds, with a final extension step of 72°C for 2 minutes. Amplicons were purified with AMPure XP beads (Beckman Coulter, Indianapolis, IN) at a 0.8:1 ratio (beads:DNA) to remove primer dimers. Eluted DNA was quantitated on a Qubit fluorimeter (Life Technologies, Grand Island, NY). Sample pooling was performed on ice by combining 40 ng of each purified band. For negative controls and poorly performing samples, 20 µL of each sample was used. The sample pool was purified with the MinElute PCR purification kit (Qiagen, Germantown, MD). The final sample pool underwent 2 more purifications: AMPure XP beads to 0.8:1 to remove primer dimers, and a final cleanup in Purelink PCR Purification Kit (Life Technologies). The purified pool was quantified in triplicate on the Qubit fluorimeter prior to sequencing.

The sequencing pool was prepared according to Illumina protocols (Illumina, Inc., San Diego, CA), with an added incubation at 95°C for 2 minutes immediately following the initial dilution to 20pM. The pool was then diluted to a final concentration of 7pM + 20% PhiX control (Illumina). Sequencing was done on an Illumina MiSeq 500‐cycle V2 kit (Illumina).

*Quality control and taxonomic identification*

Sequences from the Illumina MiSeq were deconvolved and then processed through the CMM in‐house sequence quality control pipeline, which includes dust low complexity filtering, quality value (QV<30) trimming, and trimming of primers used for 16S rRNA gene amplification, and minimum read length filtering. Using the scripts fastq_quality_trimmer and fastq_quality_filter from Hannon’s Cold Spring Harbor Laboratory’s FASTAX-Toolkit reads were trimmed until the QV was 30 or higher. Trimmed reads shorter than 75bp or those with less than 95% of the bases above a QV of 30 were discarded. Forward and reversed paired reads were merged with a minimum required overlap of 25 bp, proportion overlap mismatch > 0.2, maximum N’s allowed = 4, and a read length minimum of 125 bp. Forward and reverse reads were merged into contigs then processed through the CMM’s Mothur‐based (v1.44.1) 16S rRNA gene sequence clustering and annotation pipeline (*16*). Sequence taxonomic classifications were performed with the Ribosomal Database Project’s (RDP) naïve Bayesian classifier with the SILVA 16S rRNA database (v138) (*17, 18*)

***Supplemental Figures:***

Supp. Fig. #1: All associations from Model 1 (CaaR). Each of the 25 most prevalent taxa as well as covariates are represented as predictors. Associations with individual comorbidities are demonstrated with the direction of association illustrated and P-values reported in blue.


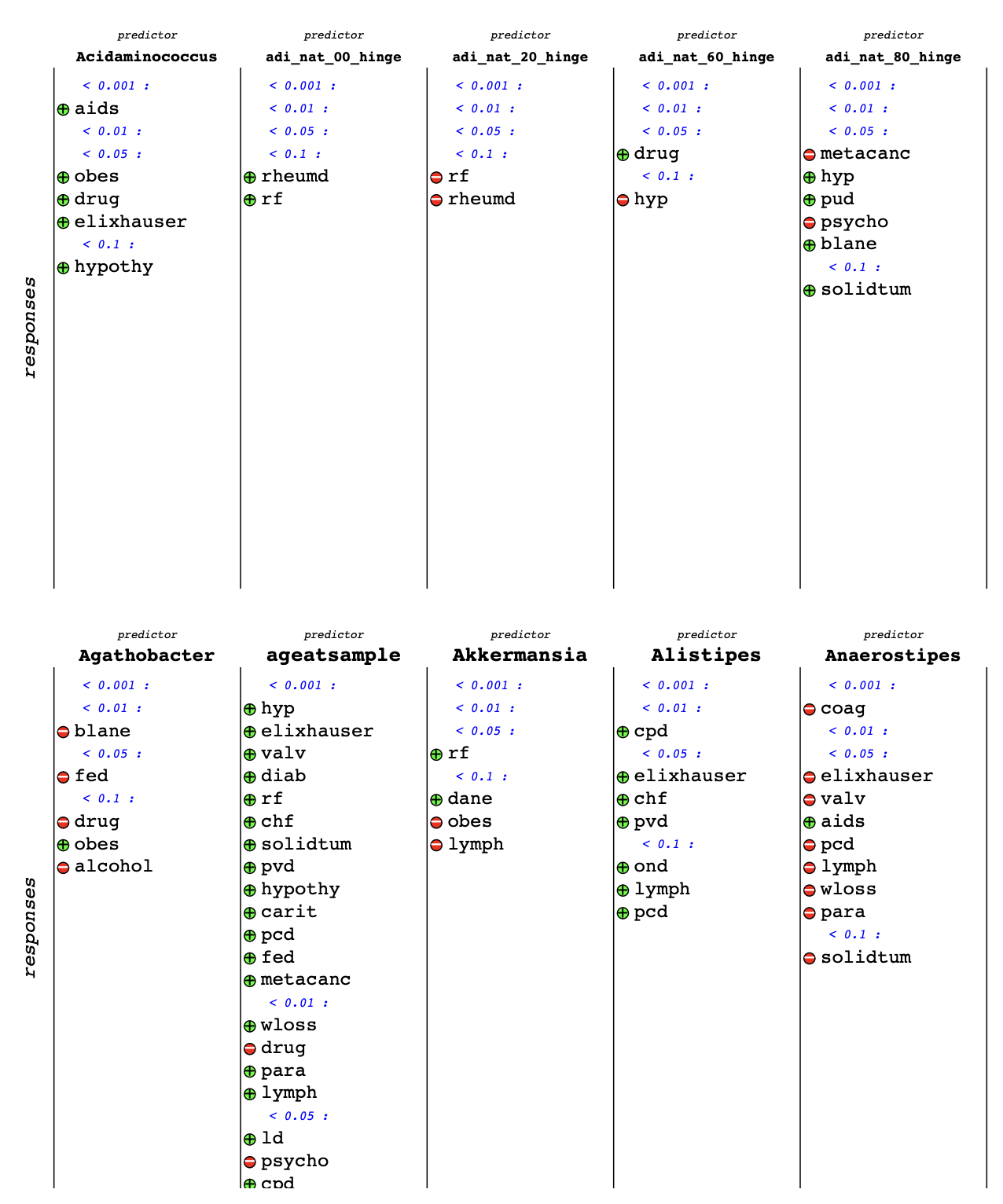


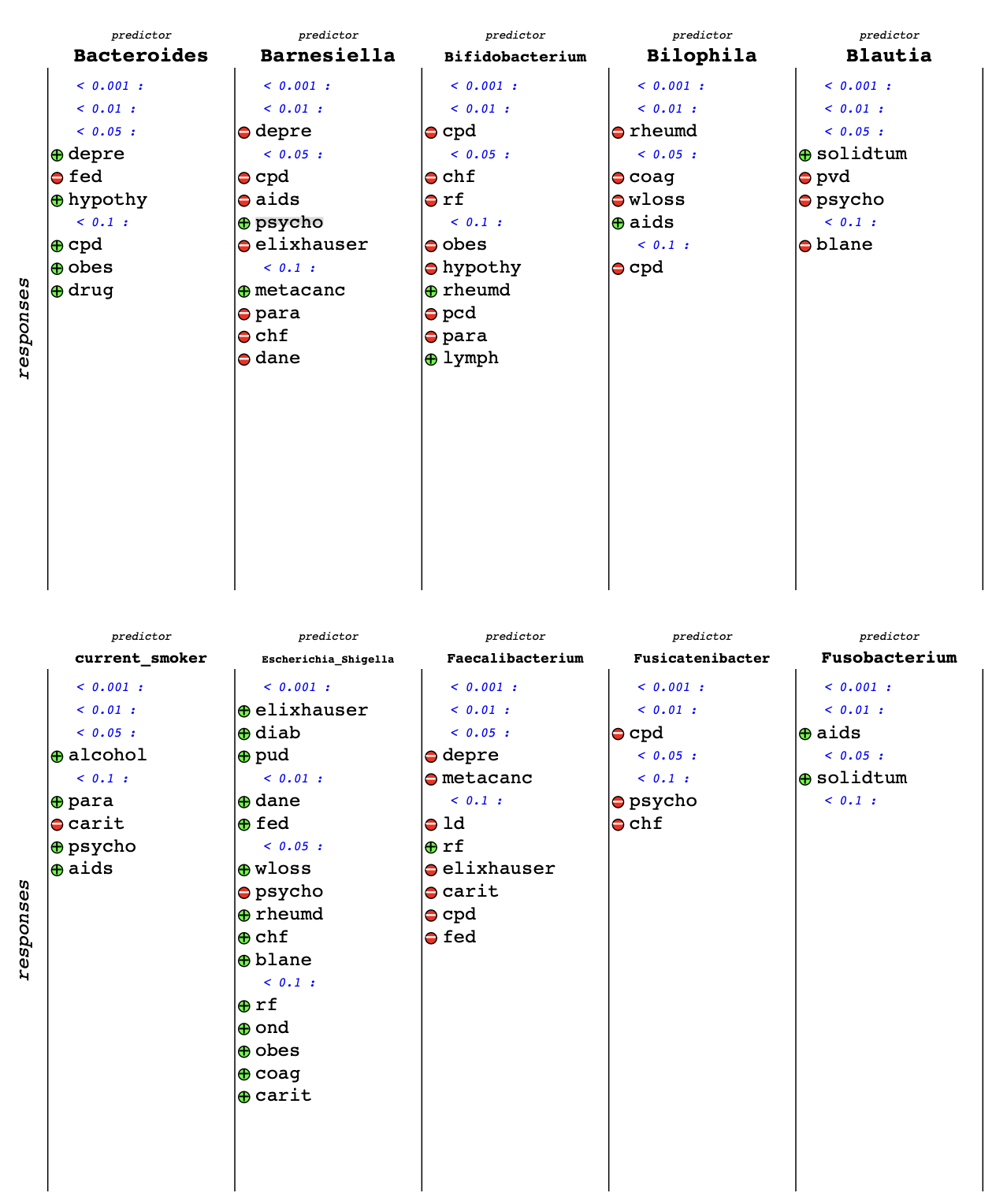


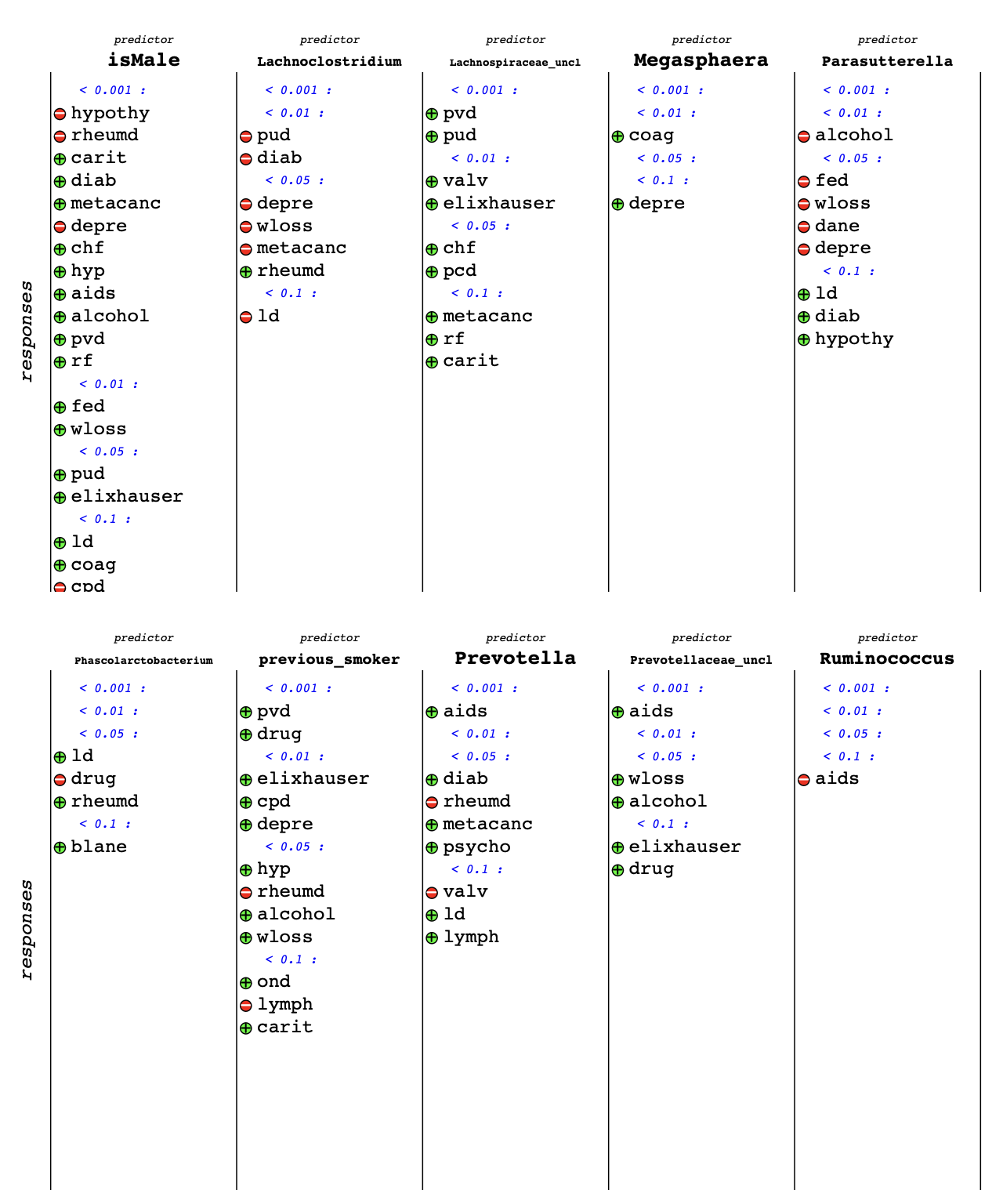

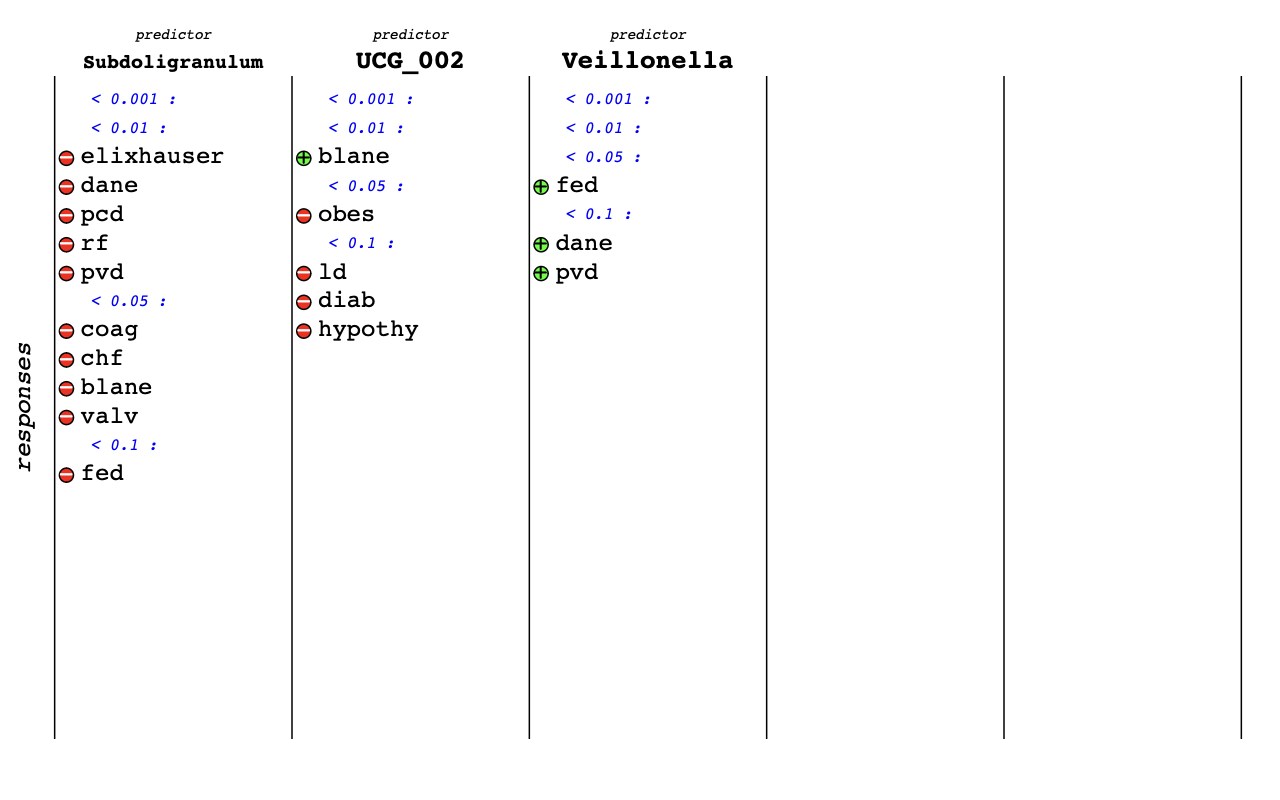


Supp. Fig. #2: Significant associations between alpha diversity and comorbidities in Model 1 (CaaR). Direction of association is illustrated and P-values are reported in blue.


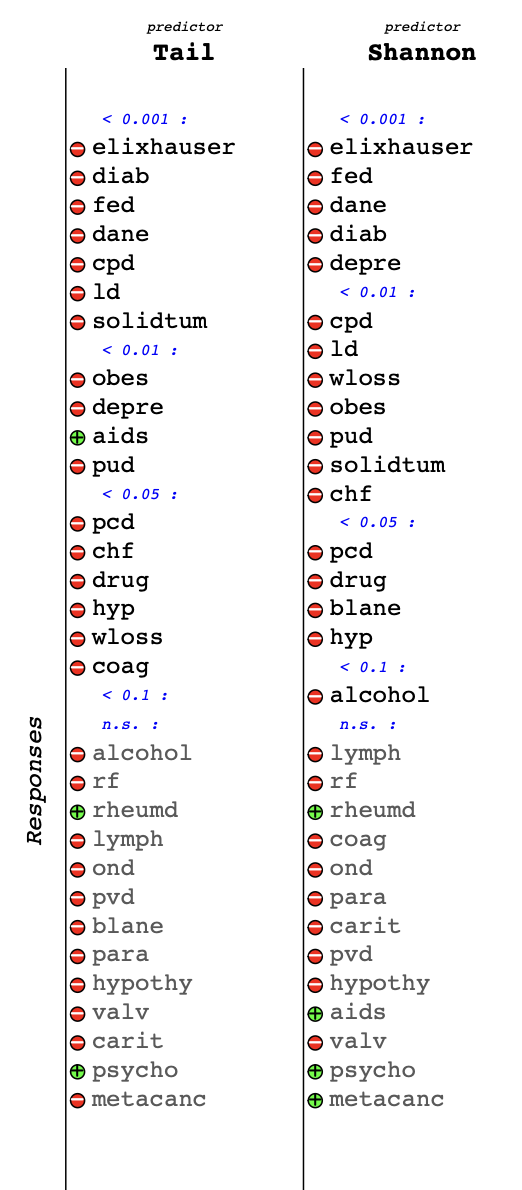

Supplement: Supplementary file 1 [file Data_Sheet_1.docx]
